# Effects of cropland-to-orchard conversion on soil multifunctionality, particularly nitrogen cycling in the eastern Loess Plateau

**DOI:** 10.3389/fmicb.2024.1471329

**Published:** 2024-10-24

**Authors:** Zhuanzhuan Fan, Jiali Wang, Dandan Lv, Shangbin Li, Yuan Miao, Mengjun Hu, Donghui Wu, Fengying Liu, Dong Wang

**Affiliations:** ^1^International Joint Research Laboratory of Global Change Ecology, School of Life Sciences, Henan University, Kaifeng, China; ^2^Henan Dabieshan National Field Observation and Research Station of Forest Ecosystem, Henan University, Kaifeng, China; ^3^School of Landscape Architecture, Beijing Forestry University, Beijing, China; ^4^Laboratory of Resources and Applied Microbiology, School of Life Sciences, Henan University, Kaifeng, China

**Keywords:** enzyme activity, pomegranate trees, soil multifunctionality, land use change, the Loess Plateau

## Abstract

The conversion of cropland to orchards is one of the main measures of the Grain for Green Program for soil and water conservation and ecosystem function maintenance in the eastern Loess Plateau, China. However, the patterns and influencing forces of soil multifunctionality during the conversion from cropland to orchard remain unclear. This study evaluated the responses and regulating factors of soil multifunctionality following the conversion of cropland to pomegranate (*Punica granatum* L.) orchard along a 10-year chronosequence. Results showed that the conversion of cropland to pomegranate trees significantly increased the L-leucine aminopeptidase enzyme activity from 4.77 to 17.69 nmol g^−1^ h^−1^. The 10-year pomegranate stand exhibited the highest nitrogen (N) cycle multifunctionality. The N cycle multifunctionality was positively correlated with soil dissolved organic carbon (C) content, soil available phosphorus content, microbial biomass C content, phospholipid fatty acid, and soil feature index (All *p* < 0.05). Structural equation modeling suggested that the increased N cycle multifunctionality was attributed to soil feature index rather than soil microbial C content and phospholipid fatty acid. Land-use change did not affect soil C cycle, phosphorus cycle, or soil multifunctionality. Overall, our findings reveal that cropland conversion to orchards significantly enhances soil N cycle multifunctionality, highlighting the soil feature index’s role in maintaining soil function. The conversion from cropland to orchards, which has economic benefits and increases soil N cycle multifunctionality, is an effective approach of the Grain for Green Program in the Loess Plateau.

## Graphical abstract

Pomegranate forest stand age increased soil nitrogen cycle multifunctionality through soil physicochemical properties rather than microbial community.
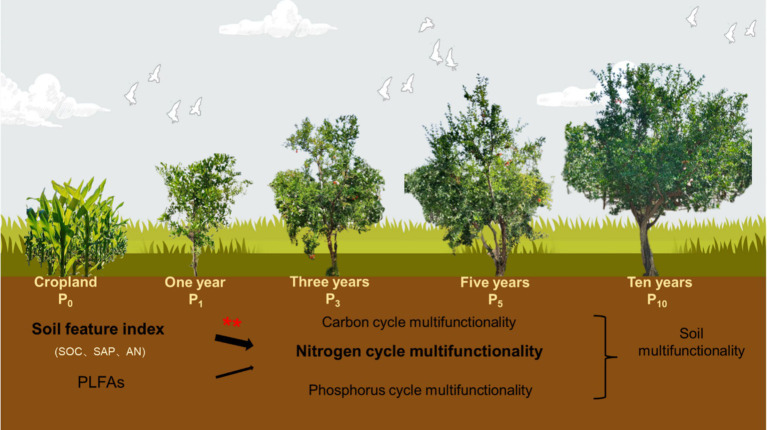

## 1 Introduction

Since 1999, the “Grain for Green” Program, i.e., converting degraded land to plantations, secondary forests, and grasslands, has been implemented in the Loess Plateau, China, to prevent soil erosion, enhance soil nutrient content, and preserve the multiple soil functions (Wang et al., 2014; Liao et al., 2016). The “Grain for Green” project and other soil conservation projects have significantly enhanced ecosystem services, including carbon sequestration and net primary production on the Loess Plateau (Zhang et al., 2023). Ecological restoration projects are the effective strategy to enhance the local ecological environment and ecosystem service functions (Li et al., 2023). Soil multifunctionality refers to the ability of an ecosystem to provide a variety of soil functions and services simultaneously, including maintaining system health, productivity and sustainability, and nutrient cycling (Delgado-Baquerizo et al., 2020; Li et al., 2022; Yang et al., 2023; Jia et al., 2024). The conversion of cropland to orchards, a key approach in the “Grain for Green” project, has economic benefits and can conserve soil and water (Liao et al., 2018). However, the effect of conversion of cropland to orchards on the soil multifunctionality remains unclear, limiting the accurate assessment of ecological and environmental benefits of the “Grain for Green” project (Daelemans et al., 2023). Thus, it is necessary to investigate the effects of the conversion from cropland to orchard on soil multifunctionality to aid in effectively assessing the ecological recovery in the Loess Plateau.

Soil multifunctionality, serves as a practical indicator for assessing the interplay among various soil environmental factors, which respond sensitively to land use changes within agroecosystems (Delgado-Baquerizo et al., 2020). Soil enzymes, as the primary drivers of soil nutrient cycling, are key indicators of soil functioning and are often used to quantify soil multifunctionality (Lei et al., 2023). Note that standing age exerts dramatic but inconsistent influences on the enzyme activity associated with multifunctionality (Luo et al., 2017). Multifunctionality increases significantly with the successional stage in the natural secondary forests of the Wuyi Mountains, China (Shi et al., 2021). Soil multifunctionality increased by 3, 8, and 10% after 6, 15, and 30 years of restoration in the riparian zones of sugarcane production landscapes, respectively (Bieluczyk et al., 2023). Furthermore, compared to abandoned cropland, natural vegetation succession led to a fivefold increase in soil multifunctionality after 35 years of agricultural restoration in Russia (Ovsepyan et al., 2020). Phosphorus cycling multifunctionality has been observed to increase with stand age across the Franz Josef time series, while carbon and nitrogen cycle multifunctionality decreased with age in the organic soils (Allison et al., 2007). In addition, multifunctionality initially increases and subsequently decreases over time in tea plantations in southeastern China (Xue et al., 2006). Despite numerous experiments conducted on land use change in the agriculture ecosystem, the impacts of stand age on the soil multifunctionality of the conversion from cropland to orchards are uncertain. Therefore, examining the responses of soil multifunctionality to stand age is required to accurately assessing the factors influencing soil functions in orchards.

Land use change plays a critical role in ecosystem services and human well-being, and affects soil multifunctionality through multiple pathways (Allan et al., 2015; Yang et al., 2023). Soil microbes are essential components of the Earth’s biodiversity and significantly affect the element cycling and nutrient supply (Allison et al., 2007; Vázquez et al., 2020). Land use change regulates soil multifunctionality by alterations in soil microbial community structure and diversity (Smith et al., 2015). Yang et al. (2023) also showed divergent responses of belowground multitrophic organisms to land-use changes, and the potential role of microbial modules in forecasting soil multifunctionality. This is effectively assessed through the analysis of phospholipid fatty acids, a key indicator of microbial biomass and composition (Shi et al., 2021). It has also been shown that land use change improves soil multifunctionality by increasing the complexity of the soil polytrophic network on the Loess Plateau (He et al., 2023). Furthermore, soil nutrients, in addition to soil microbial diversity, are also regulating soil multifunctionality (Nazaries et al., 2021; Yang et al., 2024). Soil available nutrients and soil feature index are the main drivers of soil multifunctionality in the North China Plain (Jia et al., 2022). Soil multifunctionality is driven by a combination of soil nutrients and microorganisms, with the interaction of these factors explaining a greater variation in soil multifunctionality than any single factor alone (Wang et al., 2022; Wang et al., 2023). Overall, these findings indicate that the effects of land use change on soil multifunctionality are complex and multifaceted, which are likely to be determined by soil nutrients and microorganisms (Lei et al., 2023). Therefore, it is critical to investigate the relative roles of soil nutrient features and microbial attributes in the response of soil multifunctionality to land use change.

Pomegranate (*Punica granatum* L.) production reached 1.7 million tons in China in 2017. Pomegranate is widely cultivated in the Loess Plateau due to the abundant sunshine, good ventilation, and significant diurnal temperature differences (Wang S. L. et al., 2021). Planting pomegranates not only inhibits soil erosion and promotes soil fertility but also improves soil functions (Liao et al., 2018; Zheng et al., 2022). However, studies on how long-term stand age affects soil multifunctionality in pomegranate orchards are limited, especially in the semi-humid region of the Loess Plateau. To close these knowledge gaps, we selected four orchards of different stand ages (1, 3, 5, and 10 years) and cropland to comprehensively analyze soil nutrients, enzyme activities, microbial communities, and soil multifunctionality. This study aimed to address two questions: (1) How did soil multifunctionality respond to pomegranate stand age? and (2) What are the underlying mechanisms of soil multifunctionality in response to pomegranate stand age? Our findings would contribute to the understanding of the response of soil multifunctionality to conversion of cropland to orchards and its mechanisms, which in turn would benefit orchards development.

## 2 Materials and methods

### 2.1 Site description

**Figure 1:**
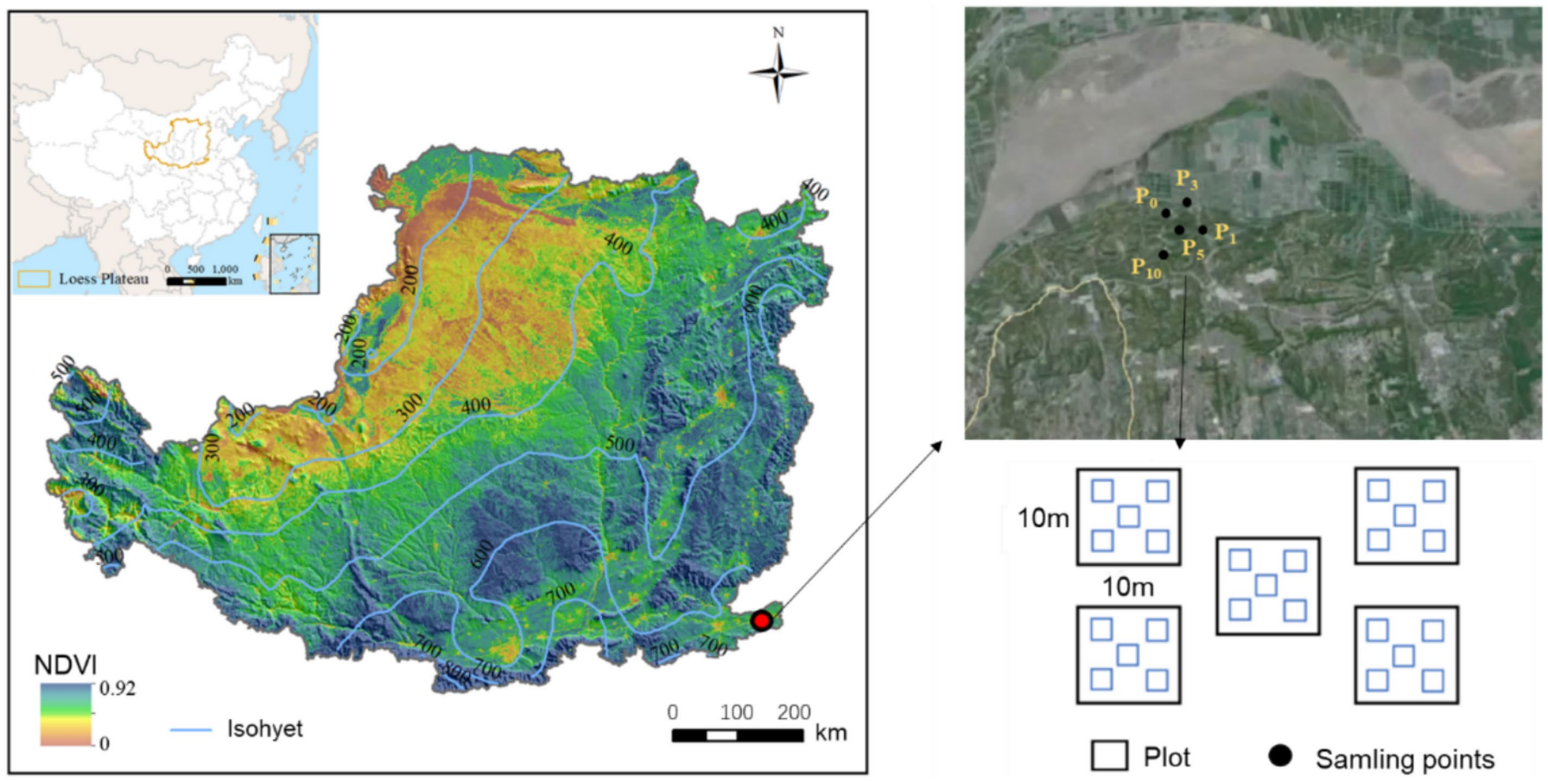
Experimental sitemap illustrating the location in the eastern part of the Loess Plateau, China. Black dots represent sample sites of the control (P_0_), pomegranate plantation of 1 year (P_1_), 3 years (P_3_), 5 years (P_5_), and 10 years (P_10_).

This study was conducted in the eastern Loess Plateau (34 °59′ 65′′ N, 113 °25′ 05′′ E, 100 m a.s.l), in Henan Province, China (Figure 1). The region has a temperate continental monsoon climate. According to the data from the China National Meteorological Information Center from 2010 to 2020, the average annual temperature was 14.3°C, with January temperatures dropping as low as −9.6°C and July reaching up to 42.5°C. The average annual precipitation was 631.4 mm, of which 65% occurred from July to September. The main soil type was classified as sandy soil. The main crops cultivated are wheat (*Triticum aestivum* L.) and maize (*Zea mays* Linn).

### 2.2 Experimental design

We selected five sites for the experiment, namely, cropland as a control (P_0_), and pomegranates with a stand age of 1 year (P_1_), 3 years (P_3_), 5 years (P_5_), and 10 years (P_10_). Five 10 m × 10 m plots were selected at each site, and the distance between two plots was larger than 100 m to ensure the independence of every plot. Each treatment included five repetitions. A single farmer owned the entire study site and applied consistent management practices across the area. These practices included uniform irrigation frequency and rate, the application of 1.5 t ha^−1^ of inorganic fertilizer and 3.0 t ha^−1^ of organic fertilizer inputs annually, and the implementation of no-tillage and weed control measures.

### 2.3 Soil sampling

In September 2020, soil samples were taken and mixed in the center and four corners of each plot by a 5 cm diameter soil drilling sampler at a depth range of 0–10 cm at each site. First, visible roots and other debris were removed from the soil sample. The soil samples were then passed through a 2 mm sieve. A total of 25 mixed soil samples were stored in a − 20°C refrigerator and divided into two subsamples to measure soil physical and chemical properties and determine soil enzyme activity, respectively.

### 2.4 Laboratory analysis

The soil enzyme activities of *β*-1, 4-glucosidase (BG), β-xylosidase (BX), β-cellobiohydrolase (BCE), L-leucine aminopeptidase (LAP), β-1, 4-N-acetylglucosaminidase (NAG) and acid phosphatase (AP) enzyme activities were measured from 1 g of soil to represent carbon, nitrogen, and phosphorus cycle multifunctionality, respectively (Chen et al., 2019; Hu et al., 2023). Dissolved organic carbon content (DOC) extracted with 0.5 M K_2_ SO_4_ of fresh soil sample was measured with a total Organic Carbon analyzer (vario TOC cube, Elementar, Germany). Soil available nitrogen content (AN) was determined using automated flow injection after the extraction of soil samples with 1 M KCl (Wang et al., 2022). Soil available phosphorus content (SAP) was extracted with 0.5 M of NaHCO_3_ and measured with an ultra-violet spectrophotometer (Fiske and Subbarow, 1925). Phospholipid fatty acids (PLFAs) were measured following the method described in Bossio and Scow (1998) and were analyzed separately using a gas chromatograph with a flame ionization detector (GC6890, Agilent Technologies, Bracknell, United Kingdom). The microbial biomass carbon (MBC) and microbial biomass nitrogen (MBN) contents were estimated by the fumigation-extraction method (Brookes et al., 1985).

### 2.5 Statistical analysis

To quantify soil multifunctionality (SMF), we used a method based on the calculation of the Z-scores of six key enzymes. In particular, the magnitude of the soil function is converted into a Z score and averaged to quantify the multifunctionality index (Lucas-Borja and Delgado-Baquerizo, 2019). We used the mean of the Z-scores of BG, BX and BCE to represent carbon cycle multifunctionality (CCM), the mean of the Z-scores of LAP and NAG to represent nitrogen cycle multifunctionality (NCM), and the Z-scores of LAP to represent phosphorus cycle multifunctionality (PCM). Following this, we calculated SMF using the average of the CCM, NCM, and PCM values.

Principal component analysis was used to calculate soil feature index (SFI) with three variables, namely, DOC, AN, and SAP (Masto et al., 2008). The DOC, AN, and SAP data were standardized using Z-scores before analysis. Using principal component analysis, two principal components were generated, with our study focusing on principal component one. The weight of the principal component one was multiplied by the Z-score normalized DOC, AN, and SAP data to calculate the values of SFI for each sample.

We used one-way analysis of variance (ANOVA) and least significant difference (LSD) to test for differences in soil nutrients, enzyme activity, PLFAs and multifunctionality among the five treatments. Prior to ANOVA, we performed a Shapiro–Wilk test to ensure that the experimental data conformed to a normal distribution to meet the assumptions of the statistical analysis. Pearson correlation analysis was performed between soil nutrients, PLFAs, enzyme activity and multifunctionality. Structural equation modeling (SEM) was used to estimate the direct and indirect effects of stand age, SFI, MBC, and PLFAs on NCM, based on *a priori* expectations of reasonable causal relationships. The difference was considered significant at the 0.05 level. These statistical analyses were carried out with R Studio (version 4.2.2, R Core Team).

## 3 Results

### 3.1 Soil nutrient and microbial biomass

**Figure 2:**
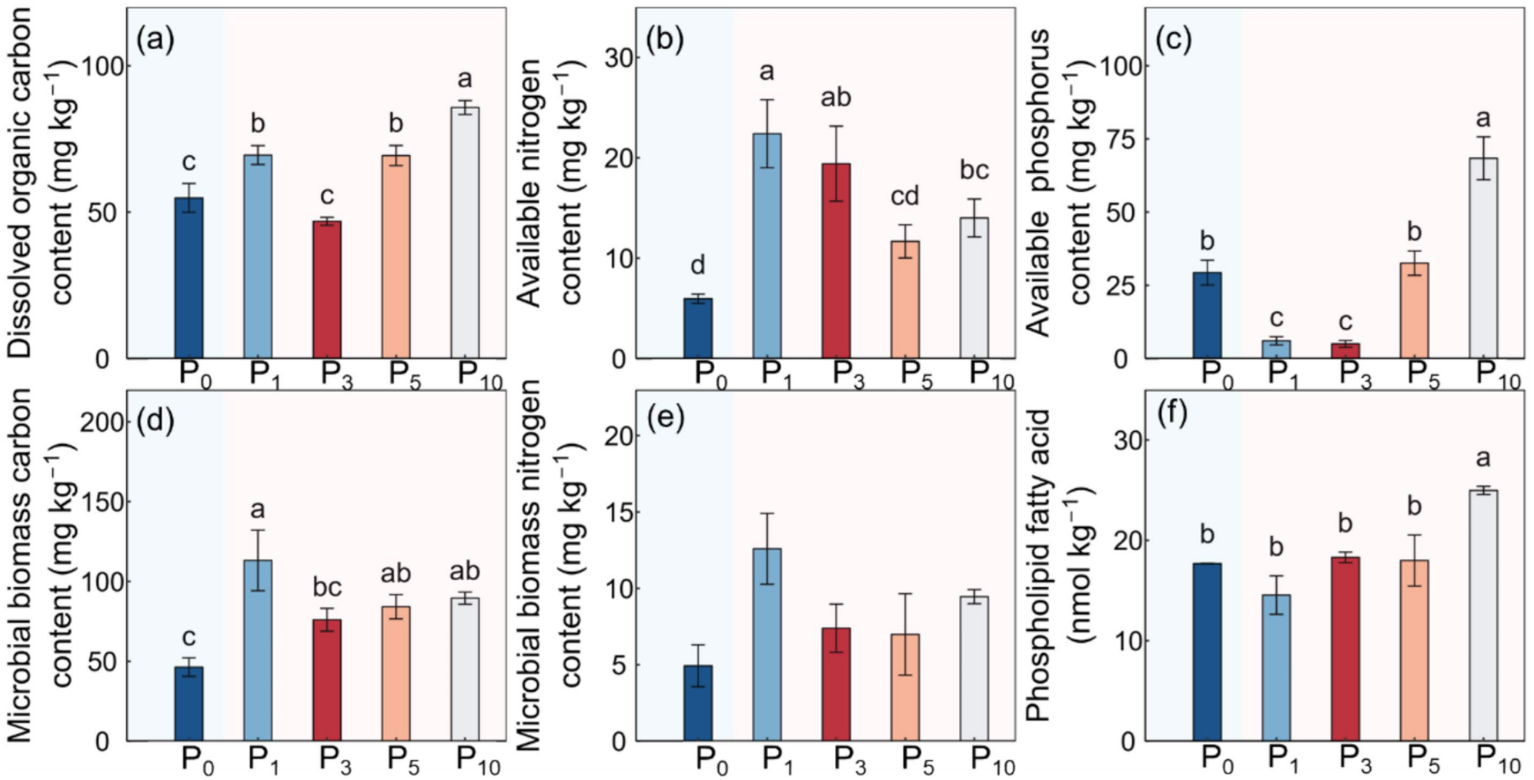
Soil dissolved organic carbon content **(a)**, available nitrogen content **(b)**, available phosphorus content **(c)**, microbial biomass carbon content **(d)**, microbial biomass nitrogen content **(e)**, and phospholipid fatty acid **(f)** under the control (P_0_), pomegranate plantation of 1 year (P_1_), 3 years (P_3_), 5 years (P_5_), and 10 years (P_10_). The data are presented as the mean ± stand error. Different letters above the error bars indicate significant differences among the five treatments at *p* < 0.05 according to LSD tests.

**Figure 3:**
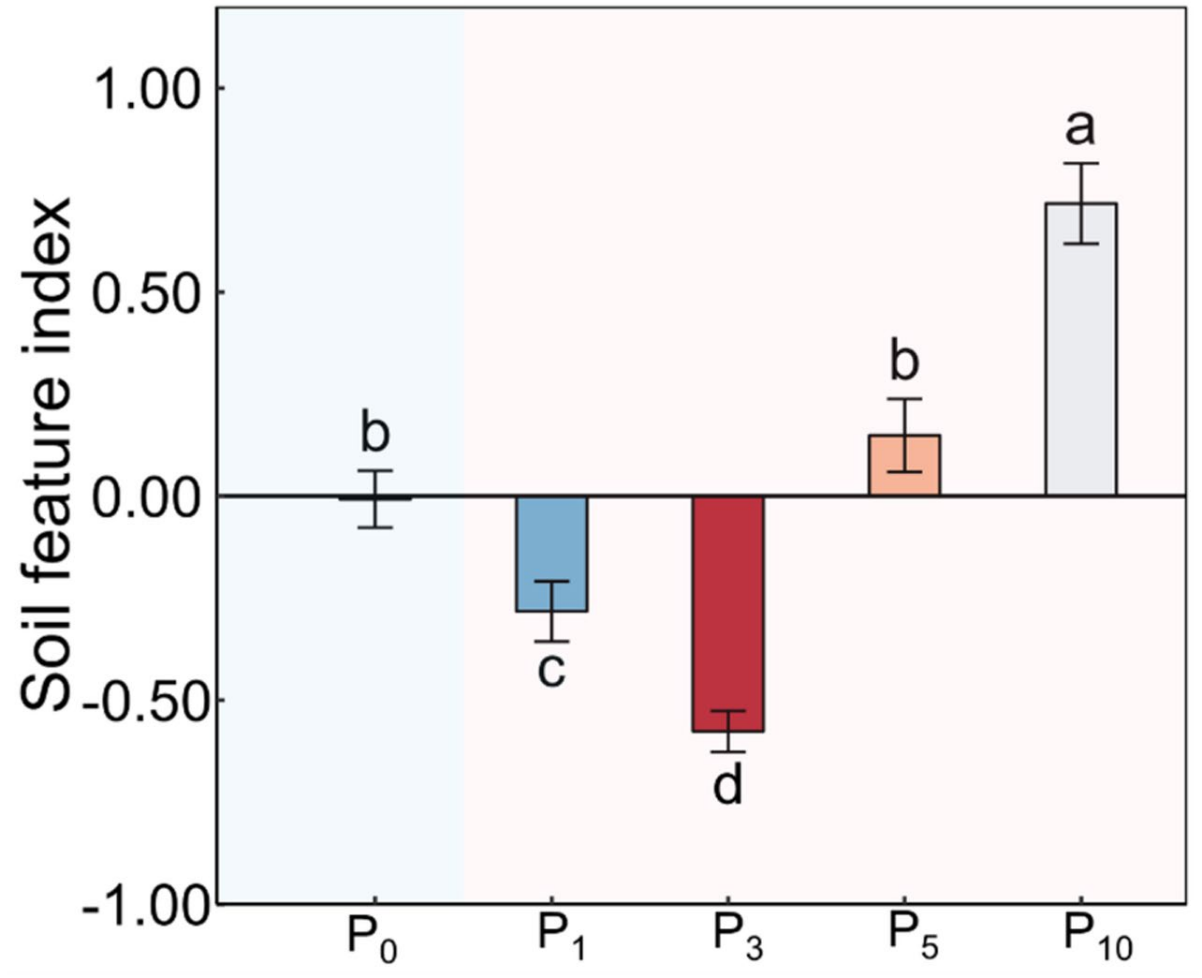
Soil feature index under the control (P_0_), pomegranate plantation of 1 year (P_1_), 3 years (P_3_), 5 years (P_5_), and 10 years (P_10_). The data are presented as the mean ± stand error. Different letters above the error bars indicate significant differences among the five treatments at *p* < 0.05 according to LSD tests.

The conversion of cropland to orchards exerted significant impacts on DOC, AN, SAP, MBC, PLFAs, and SFI (*p* < 0.05; Table 1; Figures 2, 3). The DOC of P_10_ increased by 56.2% compared to P_0_. The AN initially decreased and subsequently increased with the variations in planting time, peaking at P_1_. The SAP values first decreased and then increased after the conversion from cropland to orchards. The SAP peaked at P_10_ and increased by 133.0% compared to P_0_. The lowest MBC was observed at P_0_ and the highest at P_1_. The values of PLFAs at P_10_ were significantly higher than those at P_0_, P_1_, P_3_, and P_5_. The SFI exhibited a decreasing and subsequently increasing trend with stand ages, peaking at P_10_.

**Table 1:** Results of one-way analysis of variance (ANOVA) on the effects of pomegranate plantation age on dissolved organic carbon content, available nitrogen content, available phosphorus content, microbial biomass carbon content, microbial biomass nitrogen content, phospholipid fatty acid, and soil feature index.

Response variable	*F* value	*p* value
Dissolved organic carbon content	20.86	**<0.01**
Available nitrogen content	6.56	**<0.01**
Available phosphorus content	36.10	**<0.01**
Microbial biomass carbon content	5.67	**<0.01**
Microbial biomass nitrogen content	2.47	0.08
Phospholipid fatty acid	6.86	**<0.01**
Soil feature index	38.78	**<0.01**

The bold numerals indicate the significance at *p* < 0.01.

### 3.2 Soil enzyme activities

**Figure 4:**
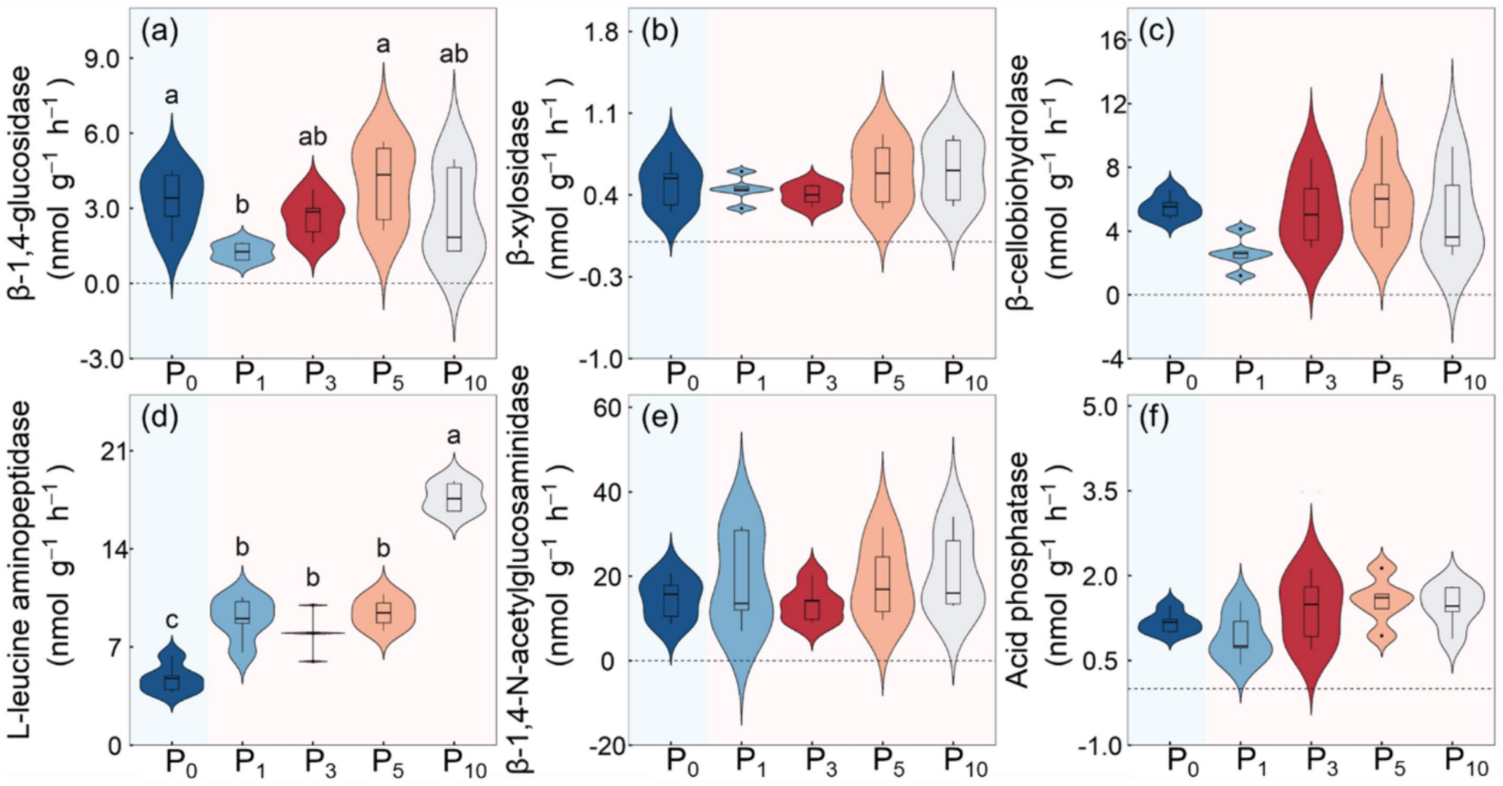
Soil *β*-1, 4-glucosidase **(a)**, β-xylosidase **(b)**, β-cellobiohydrolase **(c)**, L-leucine aminopeptidase **(d)**, β-1, 4-N-acetylglucosaminidase **(e)**, and acid phosphatasese **(f)** enzyme activities under the control (P_0_), pomegranate plantation of 1 year (P_1_), 3 years (P_3_), 5 years (P_5_), and 10 years (P_10_). The different letters indicate the significant differences among the five treatments at *p* < 0.05 according to LSD tests.

The transition from cropland to orchards significantly affected the potential activities of BG (*p* < 0.05) and LAP (*p <* 0.01), but did not affect the potential activities of BX, BCE, NAG or AP (Table 2; Figure 4). The BG was minimized under P_1_ and maximized under P_5_. The LAP was the lowest under P_0_ and the highest under P_10_, with the latter determined to be 270.9% higher than that of cropland.

**Table 2:** Results of one-way analysis of variance (ANOVA) on the effects of pomegranate plantation age on soil β-1, 4-glucosidase, β-xylosidase, β-cellobiohydrolase, L-leucine aminopeptidase, β-1, 4-N-acetylglucosaminidase, acid phosphatase enzyme activities, carbon cycle multifunctionality, nitrogen cycle multifunctionality, phosphorus cycle multifunctionality, and soil multifunctionality.

Response variable	F value	*p* value
β-1, 4-glucosidase	3.14	**0.04**
β-xylosidase	0.90	0.49
β-cellobiohydrolase	1.00	0.13
L-leucine aminopeptidase	74.57	**<0.01**
β-1, 4-N-acetylglucosaminidase	0.72	0.59
Acid phosphatase	1.75	0.18
Carbon cycle multifunctionality	1.72	0.18
Nitrogen cycle multifunctionality	9.12	**<0.01**
Phosphorus cycle multifunctionality	1.75	0.18
Soil multifunctionality	2.41	0.08

The bold numerals indicate the significance at *p* < 0.01.

### 3.3 Soil multifunctionality

**Figure 5:**
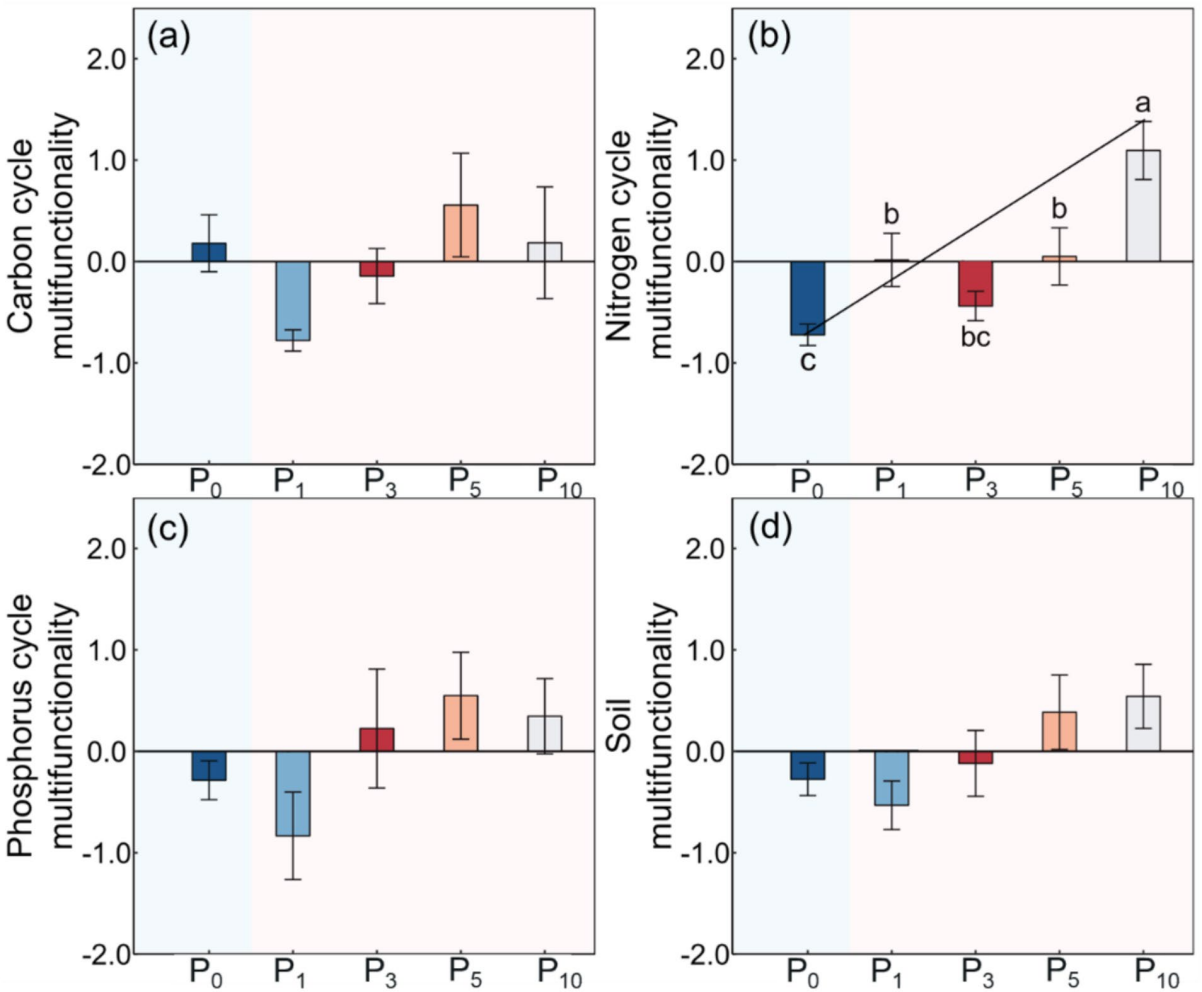
Soil carbon cycle multifunctionality **(a)**, nitrogen cycle multifunctionality **(b)**, phosphorus cycle multifunctionality **(c)**, and soil multifunctionality **(d)** under the control (P_0_), pomegranate plantation of 1 year (P_1_), 2 years (P_3_), 5 years (P_5_), and 10 years (P_10_). The data are presented as the mean ± stand error. Different letters above the error bars indicate significant differences among the five treatments at *p* < 0.05 according to LSD test.

The NCM was significantly higher after the conversion of cropland to orchards (*p <* 0.01, Table 2; Figure 5). The NCM increased with stand age and peaked at P_10_, but there was no significant difference between P_1_, P_3_, and P_5._ The CCM, PCM, and SMF were not significantly different among the five treatments.

### 3.4 Relationships between multifunctionality and soil properties, PLFAs, and SFI

**Figure 6:**
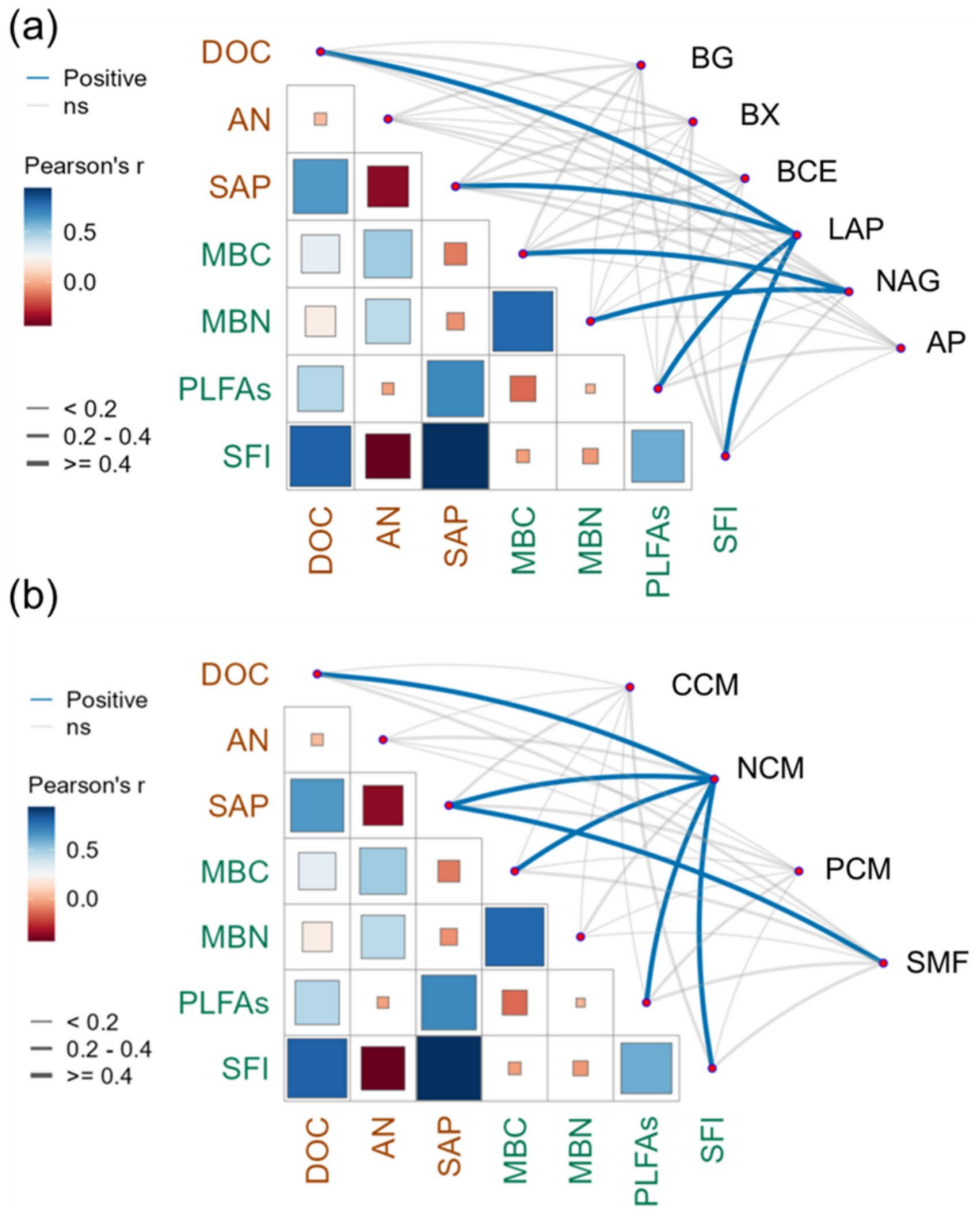
Pearson correlation matrix analysis between soil dissolved organic carbon content (DOC), available nitrogen content (AN), available phosphorus content (SAP), soil feature index (SFI), microbial biomass carbon content (MBC), microbial biomass nitrogen content (MBN), phospholipid fatty acid (PLFAs), soil β-1, 4-glucosidase (BG), β-xylosidase (BX), β-cellobiohydrolase (BCE), L-leucine aminopeptidase (LAP), β-1, 4-N-acetylglucosaminidase (NAG), acid phosphatase enzyme (AP) activities **(a)**, carbon cycle multifunctionality (CCM), nitrogen cycle multifunctionality (NCM), phosphorus cycle multifunctionality (PCM), and soil multifunctionality (SMF) **(b)**. Blue indicates a positive correlation and red a negative correlation. The depth of the color represents the r value (correlation coefficient). The thicker the line, the stronger the correlation. Blue line represents a significant positive correlation.

**Figure 7:**
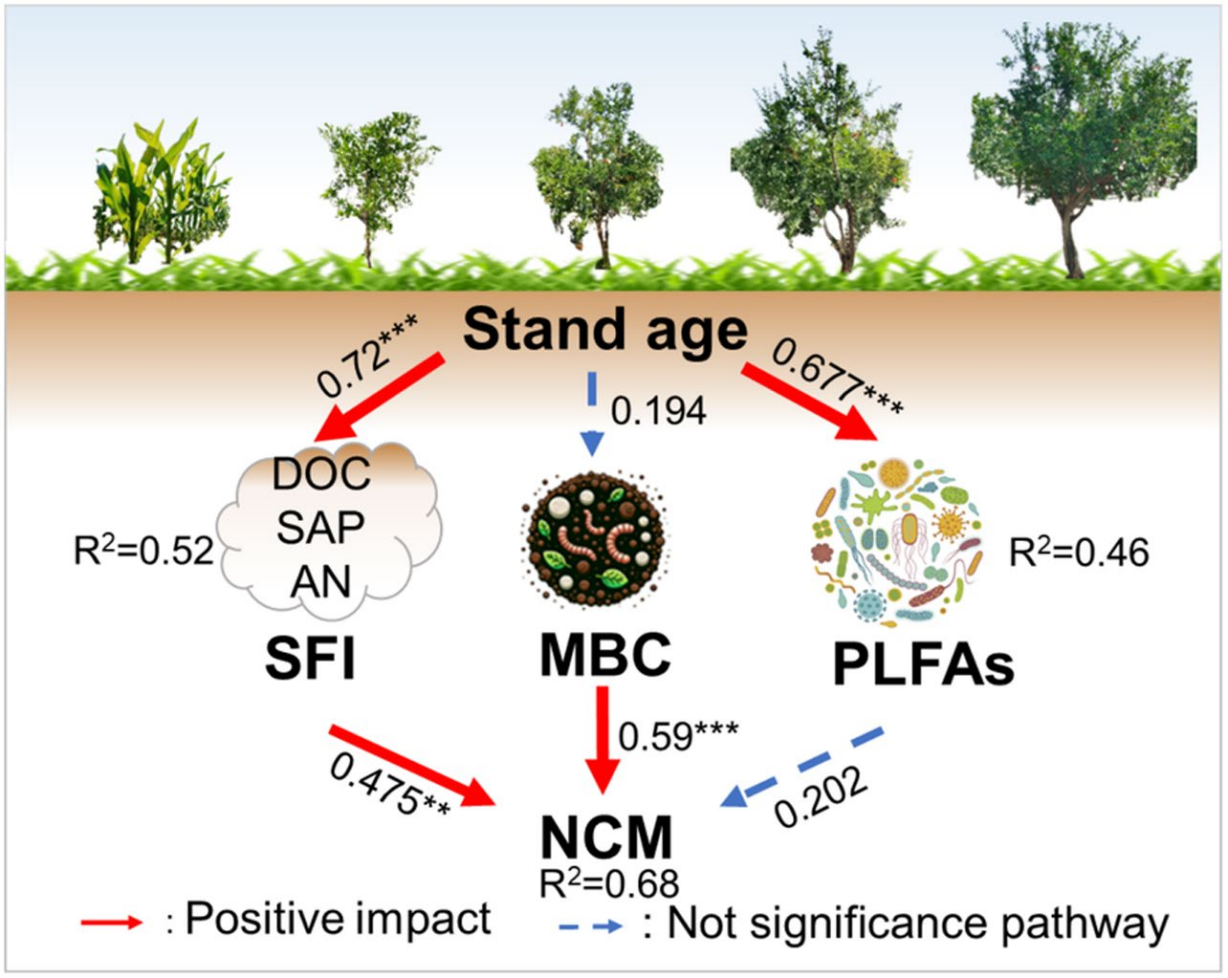
Structural equation modeling (SEM) of stand age effects on the soil feature index (SFI), microbial carbon content (MBC), phospholipid fatty acid (PLFAs) and nitrogen cycle multifunctionality (NCM). Red arrows indicate positive effects, solid arrows indicate operationally significant, and dashed arrows indicate insignificant. Arrow thickness represents the magnitude of the path coefficients; **, *p* < 0.01. ***, *p* < 0.001.

Pearson’s correlation analysis revealed that the LAP exhibited significant positive correlations with DOC (*r* = 0.78, *p <* 0.01), SAP (*r* = 0.67, *p <* 0.01), PLFAs (*r* = 0.57, *p* < 0.01), and SFI (*r* = 0.69, *p <* 0.01; Figure 6A). Moreover, the NAG presented a significant positive correlation with MBC (*r* = 0.55, *p* < 0.01) and MBN (*r* = 0.43, *p* < 0.05). The NCM was significantly positively correlated with DOC (*r* = 0.70, *p <* 0.01), SAP (*r* = 0.57, *p* < 0.01), MBC (*r* = 0.54, *p* < 0.01), PLFAs (*r* = 0.40, *p* < 0.05), and SFI (*r* = 0.57, *p* < 0.01; Figure 6B). The SMF was positively and significantly correlated with SAP (*r* = 0.43, *p* < 0.05). The SEM was performed to compare the effects of stand age on NCM in pomegranate trees (Figure 7, Fisher’s C = 13.062 with *p*-value = 0.011 and on 4 degrees of freedom). Our analysis revealed that SFI, MBC content, and PLFA pathways accounted for 68% of the overall variation in NCM. Furthermore, SFI was observed to enhance NCM rather than soil microbial (*p* < 0.01).

## 4 Discussion

### 4.1 Shifts in soil nutrients and the microbial community with stand age

Land use change alters nutrient content and microbiological properties in various ecosystems such as grassland, forest, and agroecosystems (Lucas-Borja et al., 2016; Yang et al., 2020). The DOC content increases with vegetation restoration in the semi-arid region of the Loess Plateau (Deng et al., 2019). However, our study showed that DOC decreases and subsequently increases with age. This discrepancy might be explained by regional climatic differences, as the higher rainfall in the semi-humid region of our study site compared to the semi-arid regions in other studies may lead to distinct patterns of soil nutrient dynamics (Wang et al., 2023). Increased rainfall can enhance leaching of organic carbon in the early stages of vegetation restoration, reducing DOC levels, while in later stages, improved plant growth and microbial activity may replenish soil DOC, contributing to its eventual increase (Deng et al., 2019). The AN and AP contents decreased and then increased after the conversion to orchards, which is consistent with the results along the vegetation restoration gradient in the Loess Plateau (Deng et al., 2019). Pomegranate trees affect soil properties by influencing litter, inter-root secretions, and fertilizer accumulation (Zheng et al., 2017). Compared to cropland, soil microorganisms and soil organic matter in pomegranate orchards have gradually adapted to their environment, leading to an increase in the rate of nitrogen decomposition and release (Yang et al., 2020). The observed trends of DOC and AN contents can be attributed to the intensified competition among plants and the increased demand for essential nutrients during the mid-growth period of pomegranates (Lei et al., 2023). Soil nutrient content was highest in the 10-year orchard, probably linked to the enhancement in litter inputs and nutrient cycle function (Wang et al., 2022). When pomegranate trees mature, their nutrient demand decreases as tree growth slows down, and plant residues return to the soil, thereby increasing soil nutrient content (Wang C. Q. et al., 2021). Furthermore, stopping tillage may help soil organic carbon content rise by lowering soil disturbance and soil organic carbon mineralization, which results in a rise in DOC content (Liao et al., 2018). The SFI increased significantly with the age of the pomegranate orchards, which is consistent with the study on natural forest ecosystems in Spain (Lucas-Borja et al., 2016). Mature stands typically have more efficient nutrient cycle systems that contribute to soil fertility, which in turn affects soil feature index (Wang et al., 2022). SFI refers to the capacity to sustain biological productivity, maintain environmental quality, and support nutrient cycling (Wang D. et al., 2021). Our results revealed that conversion from cropland to orchards increased the available nutrients and SFI, which were related to the responses of plant and soil properties to land use change.

Soil microbial communities not only constitute the largest part of global biological diversity, but also play a central role in the C, N, and P cycles (Zhang et al., 2017; Liao et al., 2018). The DOC content is the principal factor influencing soil microbial community diversity, suggesting a close relationship between soil microbes and soil nutrients (Yao et al., 2006). Soil microbial biomass and activity are sensitive indicators of the SFI and are crucial to the soil function of orchards (Qiang et al., 2020). The MBC increased with the stand age, which was consistent with the result obtained in one vegetation restoration process in central China (Wang et al., 2018). This increase is mainly attributed to the large amounts of litter produced by deciduous species, which enhances microbial metabolism and self-synthesis (Lu et al., 2022). In our study, PLFAs increased with the pomegranate stand age. The increase in PLFAs was attributed to increased soil nutrient availability, particularly at the initial stage (Zhong et al., 2018), reflecting the close relationship between the soil microbial community and the soil nutrient environment.

### 4.2 Shifts in NCM with stand age

The long-term presence of vegetation plays a crucial role in promoting soil functions related to soil nutrients (Wang et al., 2023). The enzyme activities involved in the nitrogen cycle remain relatively stable as tree age increases in the sloping citrus orchards of southwestern China (Qiang et al., 2020). In contrast, we observed the enzyme activities of the nitrogen cycle continue to increase with the plantation time. This difference could potentially be attributed to the distinct plant species and ecosystem types under investigation (Weand et al., 2010; Lucas-Borja et al., 2016). In addition, soil texture and topographic factors affect soil enzyme activity by altering temperature and humidity (Lu et al., 2022; Ma et al., 2022). The NCM significantly increases with the stand age, which is consistent with observations in apple orchards in the Losses Plateau (Wu et al., 2024) and in grassland restoration chronosequence (Guo et al., 2021). This is attributed to the fact that plant growth demands more nitrogen than what can be restored to the soil via the litter decomposition after the conversion of the cropland to pomegranate trees (Yang et al., 2020). Our findings are in line with Yang et al. (2020), who reported a gradual upward trend in NCM with time by vegetation growth. The NCM was positively correlated with DOC and SAP, suggesting that multiple abiotic factors, either jointly or individually, mediate soil nitrogen cycle multifunctionality (Jia et al., 2022). There was a significant positive correlation between NCM and SFI, indicating the key role of improving SFI in maintaining soil multifunctionality (Jia et al., 2022). In summary, improving the SFI is essential to promoting NCM.

In addition to soil nutrients, microorganisms are a major component in SMF (Guo et al., 2021; Shi et al., 2021; Li et al., 2024). Increased soil nutrient content and availability promote the proliferation of soil microorganisms, accelerate the geochemical cycle of soil nutrients, and ultimately improve soil functions (Fry et al., 2018; Vázquez et al., 2020; Ma et al., 2022). Microorganisms use energy to increase relative inputs, lifting the substrate limitation of soil enzymes and increasing enzyme activity of the nitrogen cycle (Tischer et al., 2015; Guo et al., 2021). Moreover, the NCM exhibits a significant positive correlation with both PLFAs and MBC, highlighting the vital role of microbes in maintaining soil function. Microenvironments with a higher SFI significantly stimulate microbial activity, which increases microbial abundance and diversity (Lei et al., 2023). Thereby transforming these microenvironments into hotspots for the decomposition of soil organic matter and nutrient cycle, potentially enhancing SMF by SFI (Jia et al., 2022; Wang et al., 2022).

Soil functions are driven by multiple factors, including changes in soil nutrient contents and biodiversity (Guo et al., 2021). The SEM revealed a positive direct influence of the SFI on soil NCM rather than the PLFAs. This suggests that soil NCM is primarily determined by soil nutrients rather than soil microbial community. However, microbial diversity regulates the SMF during natural secondary succession in the Wuyi Mountain, China (Shi et al., 2021). This difference could be due to the unique biological and environmental dynamics specific to the pomegranate tree population (Xu et al., 2023). Vegetation restoration reduces microbial nitrogen limitation, enhancing soil ecosystem functions. Consequently, improved nitrogen management in orchards can significantly benefit agroecosystems (Li et al., 2024).

### 4.3 Shifts in CCM, PCM, and SMF

In our study, we found that land use change and stand age did not significantly affect soil CCM. Among the key enzymes (BG, BX, and BCE), only the activity of BG was significantly influenced by stand age. This finding agrees with Wang C. Q. et al. (2021), who reported that BG activity varies with stand age in *Cunninghamia lanceolate* (Lamb.) Hook plantations. However, Qiang et al. (2020) observed that higher enzyme activities were associated with increased CCM along stand age in citrus orchards. In contrast, Wang et al. (2022) reported a decrease in soil enzyme activity with stand age over a 30-year plantation chronosequence. These differences highlight that although the activities of single enzymes such as BG differ, these differences are not sufficient to affect CCM as quantified by multiple enzymes (Qiang et al., 2020). The findings discrepancies could be attributed to cross-scale effects influenced by factors such as orchard type, soil composition, and climatic conditions (Chen et al., 2023). Additionally, our study, focusing on the early stages of the orchard, may not capture the long-term effects observed in other studies (Qiang et al., 2020). These varying results highlight the complexity of soil CCM drivers and the need for further research across different agroforestry systems.

The soil PCM increased with stand age in *Eucalyptus grandis* W. plantations in Brazil (Lino et al., 2016). However, stand age did not have a significant effect on soil PCM in pomegranate plantations. The observed differences may be due to the unique ecological conditions of the Loess Plateau, which require a longer time for the restoration of multiple soil functions (Wan et al., 2017). A similar conclusion was obtained in a previous study conducted in a Douglas fir plantation in Italy, which showed that AP activity did not reflect changes in soil development with stand age (Vittori Antisari et al., 2018). This may be due to the long stand-age period, giving time for the establishment of a new equilibrium in the soil (Lino et al., 2016). It is worth noting that these findings are based on specific areas of each stand ages, further investigations across a wider range of sites with greater stand ages are needed to confirm these results (Vittori Antisari et al., 2018).

Furthermore, stand age did not affect SMF in the pomegranate orchards in this study region. Pomegranate forests in the Loess Plateau region are not equivalent to a natural forest, as they have lower levels of vegetation complexity, nutrient content, and SMF (Shi et al., 2021; Wang et al., 2022; Li et al., 2024). The positive effect of mean annual precipitation on SMF is observed to be mediated by the soil moisture in the Tibetan Plateau (Jing et al., 2015). Restoration 30-year grassland supports twice soil function than the restoration 5-year grassland in a semi-arid temperate steppe in the Loess Plateau (Guo et al., 2021). The enhancement of SMF is a long process that requires decades to reach the next critical stage of development (Lucas-Borja and Delgado-Baquerizo, 2019). In this study, SMF showed an increasing trend in the P_3_, P_5_, and P_10_ treatments compared to the control. This may be due to the shortage of pomegranate trees, which may not have reached the tipping or turning point (Wan et al., 2017). Therefore, further investigation of older pomegranate trees is necessary to clarify the potential dynamics and patterns associated with SMF.

### 4.4 Applications and uncertainties

The conversion of cropland to orchard enhanced soil DOC content, AN content, SFI, and LAP activity (Liao et al., 2016). Overall, soil NCM improved as the pomegranate stand age progressed. In addition, the establishment of orchards not only improves SFI and NCM but also brings significant economic benefits and supports the prosperity of the local agricultural economy (Liao et al., 2018). Therefore, the conversion of cropland to orchards, a key aspect of the “Grain for Green” program, is particularly beneficial in the eastern Loess Plateau (Qian et al., 2014).

Our study found that stand age had no significant effect on CCM, PCM, or SMF, revealing an initial decrease and then a gradually increasing trend. The oldest trees in this study were only 10 years old, while the Chinese prickly ash orchard (*Zanthoxylum bungeanum* Maxim.) stands reached 35 years old (Liao et al., 2016). In addition, in a study conducted in the Mediterranean region, the maximum stand age of Spanish black pine (*Pinus nigra* Ar. *ssp. salzmannii*) forests was found to be 120 years (Lucas-Borja et al., 2016). These examples suggest that longer time periods are often required to fully capture the impact of land-use changes on soil processes. Furthermore, enzyme activity’s contribution to soil functioning varies between younger orchards and those between 20 and 40 years old (Zheng et al., 2022).

The restoration of SMF is a long process, potentially requiring more than 15 years to complete (Tian et al., 2022). Long-term monitoring is indispensable for assessing restoration success and guiding adaptive management strategies (Resch et al., 2021). Therefore, while our study provides valuable initial insights, further research is needed to evaluate the long-term impacts of land-use change on SMF, particularly with respect to larger spatial and temporal scales (Wu et al., 2024).

## 5 Conclusion

Our study provides insights into the effects of cropland conversion to orchards on soil multifunctionality in the Loess Plateau. After cropland to orchard conversion, soil nitrogen cycle multifunctionality increased with stand age. The SEM revealed that the soil feature index was the primary factor influencing soil nitrogen cycle multifunctionality, rather than the microbial community. Our results indicate a non-significant increasing trend in the carbon cycle, phosphorus cycle, and soil multifunctionality. Long-term monitoring of different stand ages in orchards is critical to accurately assessing soil multifunctionality. This finding is conducive to understanding the soil nutrients mediated nitrogen cycle multifunctionality mechanism along the stand ages in the orchard. These results are also useful for identifying the advantages of long-time orchard plantations and their impact on soil multifunctionality.

## Data Availability

The original contributions presented in the study are included in the article/supplementary material, further inquiries can be directed to the corresponding author/s.
